# The updated one-step multiplex RT-qPCR method for PRRSV classical strains, highly pathogenic strains and NADC30-like strains

**DOI:** 10.3389/fmicb.2026.1850527

**Published:** 2026-06-11

**Authors:** Zengliang Guo, He Zhang, Juan Bai, Changyou Xia, Ping Jiang

**Affiliations:** 1 College of Veterinary Medicine, Nanjing Agricultural University, Nanjing, China; 2 State Key Laboratory of Animal Disease Control and Prevention, Harbin Veterinary Research Institute, Chinese Academy of Agricultural Sciences, Harbin, China

**Keywords:** classical strain, diagnosis, highly pathogenic strain, multiplex RT-qPCR, NADC30-like strain, porcine reproductive and respiratory syndrome virus

## Abstract

**Introduction:**

PRRSV-2 prevalent strains mainly include C-PRRSV, HP-PRRSV, and NADC30-like, with the latter being the current dominant lineage. Due to the high recombination and genetic variation of PRRSV, existing RT-qPCR assays face an increasing risk of false negatives. Therefore, based on the current prevalent strain sequences, it is of great significance to update and establish a one-step multiplex RT-qPCR method that can simultaneously detect C-PRRSV, HP-PRRSV and NADC30-like.

**Methods:**

By downloading the latest prevalent strain full genome sequences from NCBI and isolating them in our laboratory, the conserved and type-specific target regions of the three strains were screened in the high-variable region of the *nsp2* gene. TaqMan MGB probes and primers were designed. After optimizing the reaction conditions, the standard curve, amplification efficiency, sensitivity, specificity, repeatability and clinical application effect of this method were evaluated.

**Results:**

The established standard curve showed a good linear relationship within the range of 1 × 108 to 1 × 10^3^ copies/μL, with a correlation coefficient R^2^ of 0.998 for all. The amplification efficiency ranged from 97.58 to 103.53%. The minimum detection limits for C-PRRSV, HP-PRRSV and NADC30-like were 10.128 copies, 8.998 copies and 8.458 copies, respectively. This method showed no cross-reaction with common porcine pathogens such as PRRSV-1, PCV2 and CSFV, and the intra-batch and inter-batch coefficient of variation was less than 2%. The positive rate of PRRSV in 588 samples was 15.5% (91/588), which was higher than 13.94% (82/588) of the reported methods. The consistency Kappa of the two methods was 0.87.

**Discussion:**

This study successfully established a one-step multiplex RT-qPCR method based on current prevalent strain sequences, which offers high sensitivity, strong specificity, and good repeatability, and can be used for rapid differential diagnosis of the three PRRSV subtypes in clinical samples, thereby supporting precise diagnosis, epidemiological monitoring, and prevention and control of PRRSV in China.

## 1 Introduction

Porcine reproductive and respiratory syndrome (PRRS) is a highly contagious disease caused by the porcine reproductive and respiratory syndrome virus (PRRSV). Its clinical symptoms mainly include reproductive disorders in sows and respiratory diseases in newborn piglets (Qiu et al., 2025). Since it was first reported in the late 1980s, PRRS has become one of the most economically damaging diseases affecting the global pig farming industry, with estimated annual losses exceeding $1.2 billion in the United States alone and millions of RMB per farm in China (Neumann et al., 2005; Russell et al., 1980; Zhao et al., 2024).

PRRSV belongs to the family of *arthrovirus* of the family of *retroviruses*. It is a single-stranded positive-sense RNA virus with a genome size of approximately 15 kb, containing at least 10 open reading frames. According to the classification standards of the International Committee on Taxonomy of Viruses (ICTV), PRRSV is divided into two genotypes: Betaarterivirus suid 1 (PRRSV-1, European type) and Betaarterivirus suid 2 (PRRSV-2, North American type). The nucleotide similarity between the two genotypes is approximately 60% (Tao et al., 2024). In 1996, the first PRRSV strain was isolated in China and was named CH-1a, marking the beginning of the epidemic of the classic American type strain (Classical PRRSV, C-PRRSV) in China(Wang et al., 2016). In 2006, a highly pathogenic PRRSV variant strain (Highly pathogenic PRRSV, HP-PRRSV) was discovered, characterized by high fever, high incidence rate and high mortality rate. The nsp2 region of this strain has a discontinuous deletion of 30 amino acids (including the deletion of leucine at position 481 and a continuous 29 amino acid deletion from position 533–561), resulting in a significant increase in virulence (Li C. et al., 2025). In 2012, a strain similar to NADC30 was first reported in China. This strain was highly homologous to the NADC30 strain isolated in the United States in 2008. The *nsp2* gene of this strain had a discontinuity of 131 amino acids (Li et al., 2012). Based on the phylogenetic analysis of the ORF5 gene, the prevalent PRRSV-2 strains in China can be divided into multiple lineages, mainly including PRRSV-2 which is mainly divided into lineage 1 (L1.8/NADC30-like and L1.5/NADC34-like), lineage 3 (L3/QYYZ-like), lineage 5 (L5.1/VR2332-like), and lineage 8 (L8.7/JXA1-like and L8/CH-1a-like) (Yim-Im et al., 2023). In recent years, the class NADC30 strain has evolved into the dominant epidemic strain in pig herds in China (Zhang et al., 2025). This strain has an extremely high recombination frequency and is prone to genetic recombination with other lineage strains, demonstrating a strong genetic evolution ability. At the same time, highly pathogenic strains and classic strains still occur sporadically in some pig farms (Yu et al., 2026). The frequent recombination characteristics of the class NADC30 strain, combined with the fact that the existing conventional vaccines cannot provide complete cross-protection, have made the genetic background of PRRSV in China increasingly complex, posing a huge challenge to the precise prevention and control of breeding enterprises.

Currently, the identification of PRRSV variants mainly relies on sequencing and evolutionary analysis based on the ORF5 or *nsp2* genes (Yu et al., 2026; Zhao et al., 2022). Although this method is accurate, it is complex to operate and time-consuming, making it difficult to meet the rapid detection requirements for clinical outbreaks (Pan et al., 2023). Compared with ORF5, the nsp2 gene contains strain-specific discontinuous deletion patterns (e.g., 30-aa deletion in HP-PRRSV and 131-aa deletion in NADC30-like strains), which provides higher resolution for differentiating the three subtypes. Fluorescence quantitative PCR technology, due to its sensitivity, specificity, and high throughput, has become an important means for PRRSV detection. Many studies have established universal or typing detection methods based on conserved genes such as ORF7 or ORF6 (Tian et al., 2025). However, as PRRSV-2 continues to mutate and recombine, the detection targets of the existing methods may no longer match the field-released strains, leading to the risk of false negatives and missed detections. Therefore, keeping up with the evolution dynamics of the field strains and regularly updating primers and probes are crucial to ensure diagnostic accuracy. For this reason, this study downloaded a large number of PRRSV sequences from GenBank and combined the whole genome data of the isolates from our laboratory. Through sequence comparison analysis, conserved and type-specific targets were screened out, successfully establishing a new one-step multiplex RT-qPCR method that can simultaneously detect NADC30-like strains, highly pathogenic strains, and classic strains, aiming to provide a reliable technical tool for the precise diagnosis, epidemiological tracking, and prevention and control decisions of PRRSV in China.

## 2 Materials and methods

### 2.1 Strains and samples

The template used in this study for constructing the plasmid standards was the RNA of the PRRSV classic strain (C-PRRSV), PRRSV-NADC30-like strain, and highly pathogenic strain (HP-PRRSV). The specific quality control samples for verification included the nucleic acids of the European type of porcine reproductive and respiratory syndrome virus (PRRSV-1), porcine circovirus type 2 (PCV2), swine fever virus (CSFV), porcine parvovirus (PRV), porcine parainfluenza virus (PPV), porcine epidemic diarrhea virus (PEDV), *Streptococcus suis* (*S. suis*), and *Glaesserella parasuis* (*G. parasuis*). All these nucleic acid samples were stored in our laboratory. From 2024 to 2026, 588 samples were collected from large-scale and medium-sized pig farms in Jiangsu Province, Zhejiang Province and other areas. These samples included nasal swabs, sera and tissues. Some of them were from pigs with respiratory symptoms, abortions, reproductive disorders, while others were from healthy pig herds.

### 2.2 Design of primers and probes

The complete genomic sequences of representative strains of PRRSV, including the classical strain (GenBank accession no. AY032626.1), the NADC30-like strain (GenBank accession no. JN654459.1), and the highly pathogenic strain (GenBank accession no. EF635006.1), were downloaded from the GenBank database. Multiple sequence alignments were performed using the Megalign software, and three highly conserved regions specific to each strain were selected from the *nsp2* gene (highly variable region) of the strains as targets. Multiple primer sets and TaqMan MGB probes were designed using the Primer Premier 5.0 software, and then the primers and probes with high scores, few primer self-dimers, primer pair dimers, and primer hairpins were selected using the PrimerSelect software (Table 1). The 5′ ends of the probes for the classical PRRSV strain, the NADC30-like strain, and the highly pathogenic strain were labeled with FAM, Cy5, and NED fluorescent groups, respectively, and the 3′ ends of the probes were all labeled with MGB quenching groups. The primers and probes were synthesized by Sangon Biotech (Shanghai) Co., Ltd. and the working concentration was 10 μM.

**TABLE 1:** Sequence information of primers and probes for identifying three strains of PRRSV.

Pathogen	Primers and probes (sequence 5′–3′)	Amplification size
C-PRRSV	F:ACCTTCCGTGAGCGCAAAG R:GCCCCTGGTTGAGCATCTC Probe:FAM-CTTACCATTGGTTCAGTAGC-MGB	110 bp
PRRSV-NADC30-like	F:ACTGCCCCCTATCAACCAGC R:GGAGTTGCCGCCCAATG Probe:Cy5-CTTCGCTGGTATTCC-MGB	181 bp
HP-PRRSV	F:TACTTGTGCCCGCGTCG R:CCAGGATGCCCATGTTCTG Probe:NED-CTGTGACAACAACGCT-MGB	192 bp

### 2.3 Preparation of recombinant plasmid standards

Using the RNA of the classical strain, the NADC30-like strain and the highly pathogenic strain as templates, the RNA was reverse-transcribed into cDNA according to the instructions of TransScript^®^ II Reverse Transcriptase (TransGen Biotech, China), and then PCR amplification was performed using the general primers listed in Table 1. Amplification system: 2 × EasyTaq^®^ PCR SuperMix 25 μL, F/R (10 μM) each 2 μL, cDNA 2 μL, Nuclease-free Water 19 μL. Amplification conditions: 94°C for 3 min, 94°C for 30 s, 55°C for 30 s, 72°C for 30 s, a total of 35 cycles, 72°C for 5 min. The amplified products were subjected to 2% agarose gel electrophoresis, purified and recovered using the gel recovery kit, and cloned into the pEASY^®^-T1 vector (TransGen Biotech, China). The transformed bacteria were picked up, single colonies were selected, and the positive plasmids with correct sequences were chosen for further expansion culture. The plasmids were extracted and their concentrations were determined. Then, the plasmid copy number formula was used for conversion (Tian et al., 2025). The standard products of the recombinant positive plasmids were named pEASY^®^-T1-C-PRRSV, pEASY^®^-T1-PRRSV-NADC30-like and pEASY^®^-T1-HP-PRRSV respectively. Plasmid (copies/mL) = (6.02 × 10^23^) × (X ng/mL × 10^–9^)/plasmid length(bp) × 660.

### 2.4 Optimization of multiple fluorescence quantitative RT-PCR reaction conditions

Using the RNA of three different strains as templates, the primer concentration, probe concentration and annealing temperature of the multiplex RT-qPCR method were optimized respectively. The primer concentration and probe concentration were set at 0.1, 0.2, 0.3, 0.4, and 0.5 μM, and the annealing temperature was set at 58 59 60, and 61°C, respectively. The group with the smaller Ct value, stronger fluorescence signal and better amplification curve was selected as the optimal reaction conditions (Wang et al., 2024). RT-qPCR amplification system: 10 μL of 2 × PerfectStart^®^ Multiplex Probe One-Step Reaction Mix, 0.8 μL of TransScript^®^ II Multiplex Probe One-Step Enzyme Mix UDG, 0.4 μL of Passive Reference Dye (50 × ), F/R at 0.1–0.5 μM each, Probe at 0.1–0.5 μM each, 1 μL of RNA, and RNase-free water to make up to 20 μL. Reaction conditions: 50°C for 5 min, 94°C for 30 s, 94°C for 5 s, 58°C/59°C/60°C/61°C for 30 s each, for a total of 40 cycles.

### 2.5 Establishment of the standard curve

The three recombinant plasmid standards were diluted in a 10-fold ratio. A gradient ranging from 1 × 10^8^ to 1 × 10^3^ copies/μL was selected as the template. Under the optimized reaction system and conditions, one-step multiplex RT-qPCR amplification was performed. After the reaction, the logarithm of the plasmid copy number was plotted on the x-axis and the Ct value on the y-axis. Each standard curve was drawn, and the correlation coefficient (R^2^) and amplification efficiency (Eff%) were calculated.

### 2.6 Sensitivity test

After mixing the three plasmid standards, they were diluted 10-fold from 1 × 10^6^ copies/μL to 1 × 10^0^ copies/μL using sterile water as the template for detection. This was to preliminarily determine the gradient that the one-step multiplex RT-qPCR could detect. On this basis, lower copy number gradients (such as 20, 10, 5, 1.25 copies/μL) were set, and each gradient was repeated for 25 detections. The detection rate was calculated, and probit regression analysis was performed using SPSS software to determine the limit of detection (LOD) of the multiplex RT-qPCR method (Wang H. et al., 2025).

### 2.7 Specificity test

Using the established one-step multiplex RT-qPCR method, the specific quality control samples were tested, including the nucleic acids of PRRSV-1, PCV2, CSFV, PRV, PPV, PEDV, *S. suis*, and *G. parasuis*. At the same time, the RNA of the classical strain of C-PRRSV, PRRSV-NADC30-like, and HP-PRRSV were used as positive controls, and RNase-free water was used as the negative control to evaluate whether this method would have cross-reactions with other porcine pathogens.

### 2.8 Repetitive tests

Three concentrations of mixed plasmid standards (high: 1 × 10^7^ copies/μL, medium: 1 × 10^5^ copies/μL, low: 1 × 10^3^ copies/μL) were selected as templates. The one-step multiplex RT-qPCR method established was used to conduct intra-batch (repeated 3 times for each concentration in the same experiment) and inter-batch (3 consecutive independent experiments) repeatability tests. The average Ct values, standard deviation (SD), and coefficient of variation (CV) of each concentration gradient were calculated.

### 2.9 Clinical application

A total of 588 clinical samples from different pig farms were collected, including oral swabs, serum, lung tissues, and lymph nodes. The nucleic acids of the samples were extracted using the MagicPure^®^ Up 32 Viral DNA/RNA Kit (TransGen Biotech, China). The samples were then tested in parallel using both the one-step multiplex RT-qPCR method established in this study and the RT-qPCR method that has been reported for detecting different PRRSV strains (Qiu et al., 2020). The positive coincidence rates of the two methods were compared.

## 3 Results

### 3.1 Reaction conditions of the one-step multiplex RT-qPCR method

**FIGURE 1:**
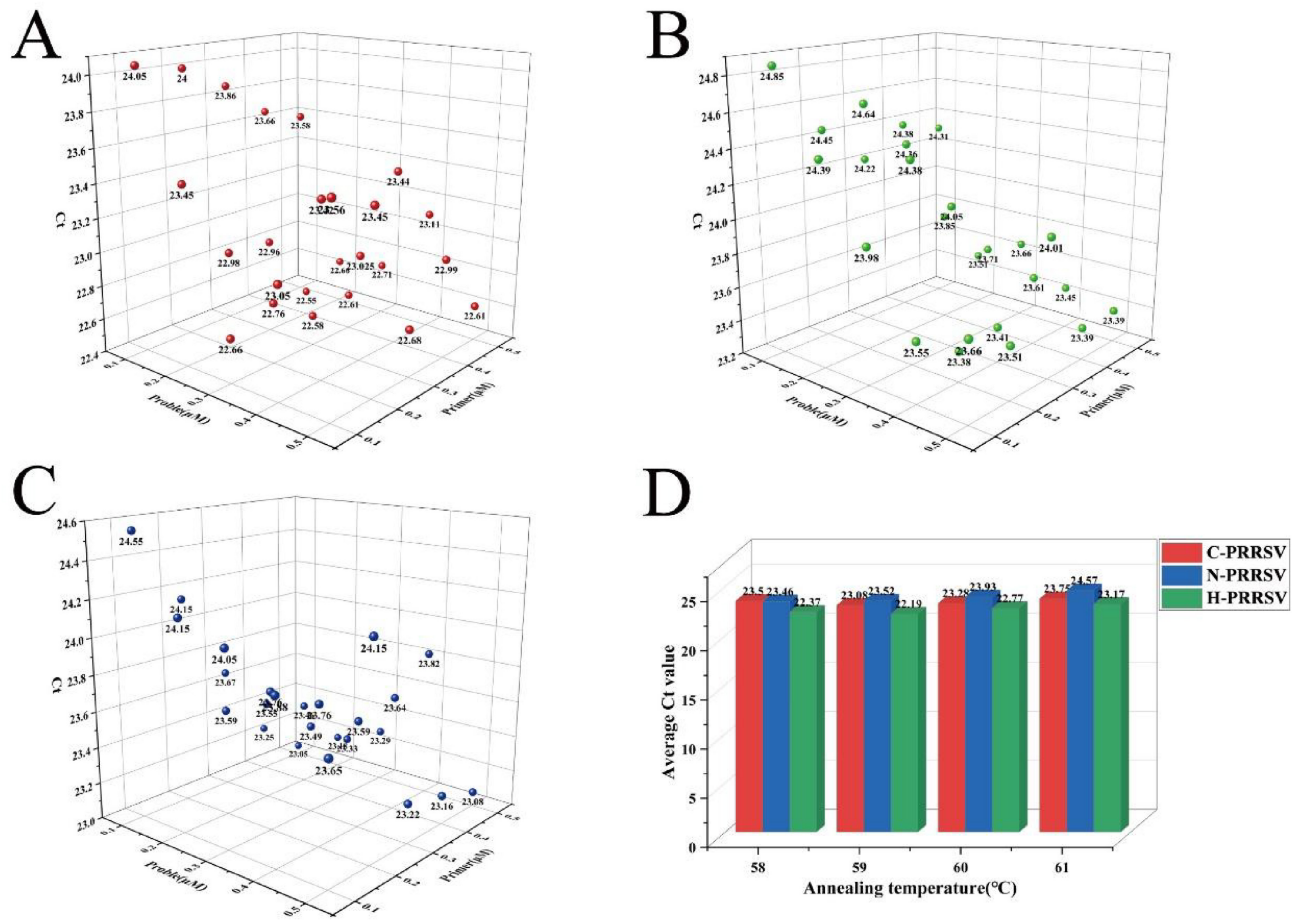
Results of optimization of reaction conditions for the one-step multiplex RT-qPCR detection method. **(A)** Optimization of primer and probe concentrations for detecting the classical strain of PRRSV. The optimal primer and probe concentrations were 0.4 and 0.2 μM, respectively. **(B)** Optimization of primer and probe concentrations for detecting the PRRSV-NADC30-like strain. The optimal primer and probe concentrations were 0.5 and 0.1 μM, respectively. **(C)** Optimization of primer and probe concentrations for detecting the highly pathogenic strain of PRRSV. The optimal primer and probe concentrations were 0.2 and 0.4 μM, respectively. **(D)** The average Ct values of the one-step multiplex RT-qPCR detection at 58, 59 60, and 61°C. When the annealing temperature was 59°C, the Ct values of the three fluorescence channels were all smaller. The error bars represent Mean Ct ± SD.

To enhance the detection performance of the multiplex RT-qPCR method, we optimized the primer, probe concentrations and annealing temperature. The results showed that when the final concentrations of the primers for C-PRRSV, PRRSV-NADC30-like and HP-PRRSV were 0.4, 0.5, and 0.2 μM respectively, and the final concentrations of the probes were 0.2, 0.1, and 0.4 μM (Figures 1A–C), and the annealing temperature was 59°C (Figure 1D), the amplification effect was better, with smaller Ct values and higher amplification efficiency. The specific reaction systems and reaction conditions are shown in Tables 2, 3.

**TABLE 2:** One-step multiplex RT-qPCR reaction system.

Component of the reaction system	Volume
2 × PerfectStart^®^ Multiplex Probe One-Step Reaction Mix	10 μL
TransScript^®^ II Multiplex Probe One-Step Enzyme Mix UDG	0.8μL
Passive Reference Dye (50 × )	0.4 μL
C-PRRSV-F/C-PRRSV-R/C-PRRSV-Probe	0.8 μL/0.8 μL/0.4 μL
PRRSV-NADC30-like-F/PRRSV-NADC30-like-R/PRRSV-NADC30-like-Probe	1.0 μL/1.0 μL/0.2 μL
HP-PRRSV -F/HP-PRRSV-R/HP-PRRSV-Probe	0.4 μL/0.4 μL/0.8 μL
RNA	1.0 μL
RNase-free Water	2.0 μL
Total	20 μL

**TABLE 3:** Conditions for one-step multiplex RT-qPCR reaction.

Reaction temperature	Time	Cyclic number
50°C	5 min	1
94°C	30 s	1
94°C	5 s	40
59°C	30 s	

### 3.2 Standard curve of one-step multiplex RT-qPCR method

**FIGURE 2:**
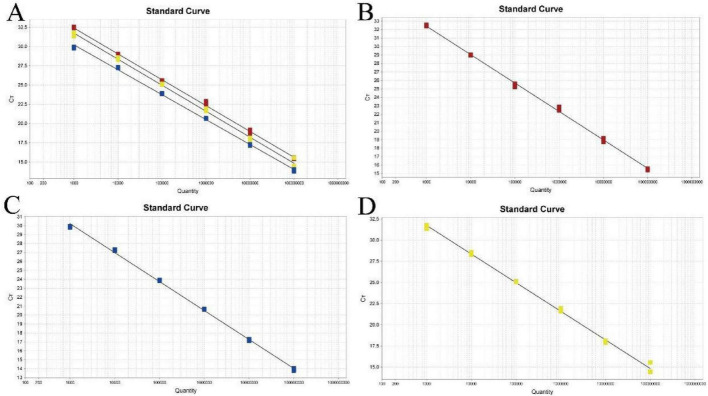
Standard curve of the one-step multiplex RT-qPCR method. **(A)** Overlay of standard curves for C-PRRSV, PRRSV-NADC30-like and HP-PRRSV. **(B)** C-PRRSV: linear equation Y = −3.361 lg(X) + 42.496, *R*^2^ = 0.998, Eff = 98.383%, Error = 0.036. **(C)** PRRSV-NADC30-like: linear equation Y = −3.240 lg(X) + 39.953, *R*^2^ = 0.998, Eff = 103.532%, Error = 0.032. **(D)** HP-PRRSV: linear equation Y = −3.381 lg(X) + 41.886, *R*^2^ = 0.998, Eff = 97.577%, Error = 0.041.

The optimized one-step multiplex RT-qPCR method was used to detect different gradient levels of mixed plasmids (each gradient was repeated for 3 detections) within the range of 1 × 10^8^ to 1 × 10^3^ copies/μL. The logarithm of the plasmid copy number was taken as the abscissa, and the Ct value as the ordinate. The results showed that the standard curves of the one-step multiplex fluorescence quantitative RT-qPCR method for detecting the three PRRSV strains within this range all had good linear relationships, with R^2^ values of 0.998, and had high amplification efficiency (Figure 2). The efficiencies were 98.383, 103.532, and 97.577%, respectively, all within the ideal amplification efficiency range (90–110%).

### 3.3 Sensitivity and minimum detection limit

**FIGURE 3:**
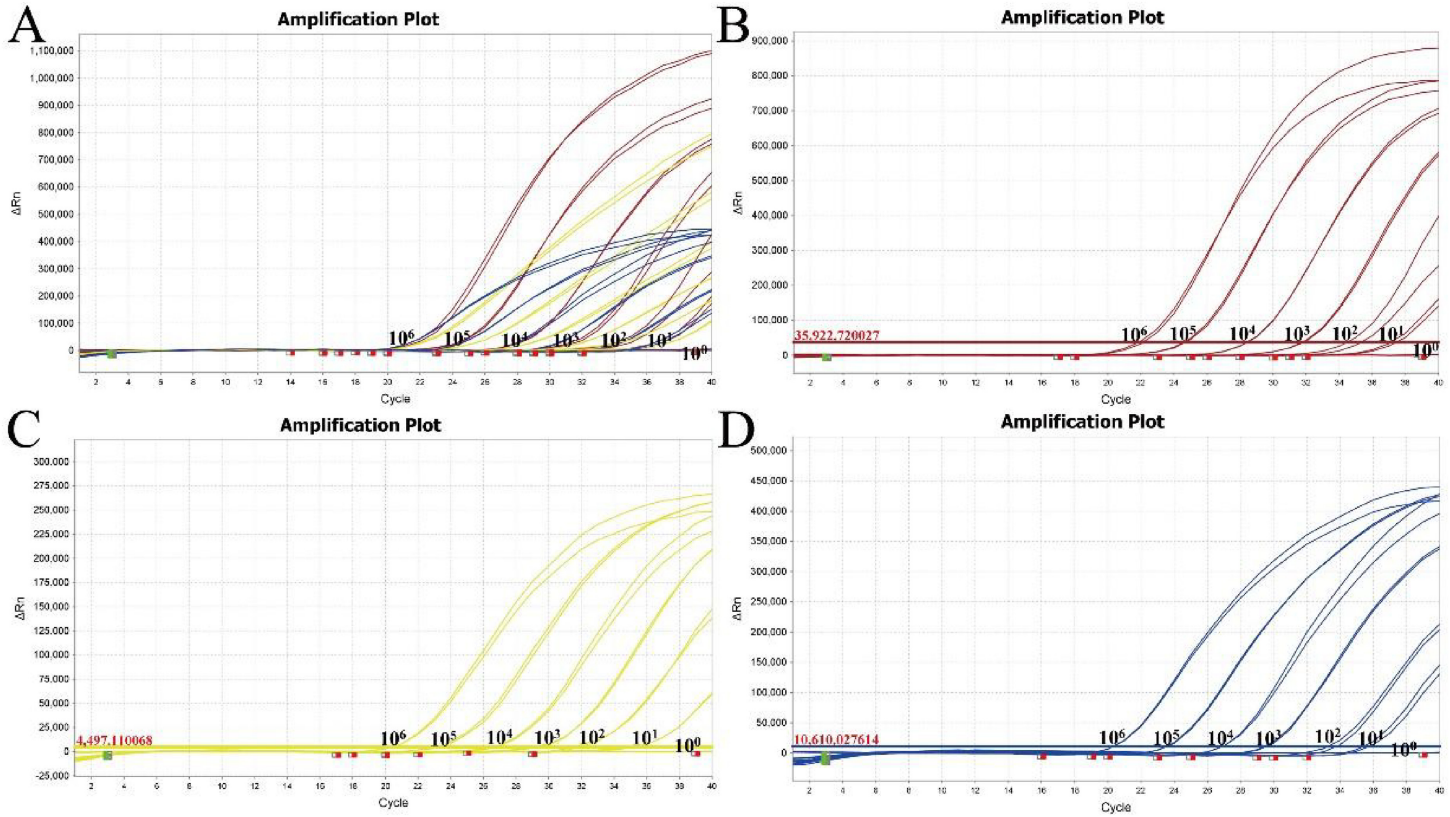
Preliminary detection results of different gradient mixed plasmids using the one-step multiplex RT-qPCR method. **(A)** Amplification curves of C-PRRSV, PRRSV-NADC30-like and HP-PRRSV using the one-step multiplex RT-qPCR method. **(B–D)** Single amplification curves of C-PRRSV, PRRSV-NADC30-like and HP-PRRSV detected by the one-step multiplex RT-qPCR method at different gradient mix ratios. The lowest copy number of the used plasmid is 10^0^ copy.

Using the one-step multiplex RT-qPCR method, different gradient mixtures of plasmids ranging from 1 × 10^6^ copies/μL to 1 × 10^0^ copies/μL were detected. The preliminary detection results showed that 10 copies could be detected in all cases (Figure 3). Further gradient dilution and Probit regression analysis indicated (Supplementary Tables S1–S4), within a 95% confidence interval, the minimum detection limits of this method for C-PRRSV, PRRSV-NADC30-like, and HP-PRRSV were 10.128 copies, 8.458 copies, and 8.998 copies, respectively, indicating that the sensitivity of the one-step multiplex RT-qPCR method established in this study was relatively high. The minimum detection limits for C-PRRSV, PRRSV-NADC30-like and HP-PRRSV were 10.128 (95% confidence interval: 9.262–11.65), 8.458 (95% confidence interval: 6.882–22.381) and 8.998 (95% confidence interval: 7.419–12.574), respectively (Supplementary Tables S1–S4).

### 3.4 Specific detection results

**FIGURE 4:**
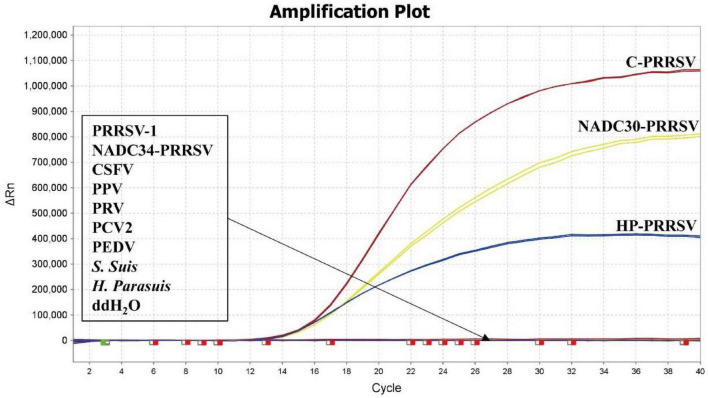
Results of specificity verification for the one-step multiplex RT-qPCR method.

Using the established one-step multiplex RT-qPCR method to amplify the specific quality control samples, the cross-reaction of this method was verified. The results showed that only the positive control wells of C-PRRSV, PRRSV-NADC30-like and HP-PRRSV exhibited corresponding specific amplification curves, while other specific quality control samples such as PCV2, CSFV, PRV, etc., and the negative control had no amplification signals (Figure 4). This indicates that this method has no cross-reaction with common porcine pathogens and is highly specific.

### 3.5 Repeatability test results

To determine the stability of the one-step multiplex RT-qPCR method we established, we conducted repetitive tests on different concentrations of plasmids at different time points. The results showed that the coefficient of variation (CV) range of intra-batch repetitive experiments was 0.18–1.71%, and the CV range of inter-batch repetitive experiments was 0.28–1.09%, all of which were less than 2% (Table 4), indicating that the one-step multiplex RT-qPCR method we established has good stability and repeatability.

**TABLE 4:** Inter-laboratory and intra-laboratory test results of one-step multiplex RT-qPCR method for high, medium and low plasmid concentrations.

Standard plasmid	Concentration of template (copies/μ L)	Intra-coefficient of variation		Inter-coefficient of variation	
		Mean Ct ± SD	CV (%)	Mean Ct ± SD	CV (%)
pEASY^®^-T1-C-PRRSV	10^7^	18.934 ± 0.034	0.18	18.551 ± 0.052	0.28
	10^5^	25.661 ± 0.099	0.39	25.438 ± 0.114	0.45
	10^3^	32.439 ± 0.211	0.65	32.518 ± 0.187	0.58
pEASY^®^-T1-PRRSV-NADC30-like	10^7^	17.428 ± 0.122	0.70	17.493 ± 0.088	0.50
	10^5^	23.662 ± 0.095	0.40	23.391 ± 0.214	0.91
	10^3^	30.429 ± 0.303	1.00	30.506 ± 0.287	0.94
pEASY^®^-T1-HP-PRRSV	10^7^	18.441 ± 0.041	0.22	18.352 ± 0.048	0.26
	10^5^	24.739 ± 0.422	1.71	24.198 ± 0.115	0.48
	10^3^	31.827 ± 0.332	1.04	31.552 ± 0.343	1.09

### 3.6 Clinical sample test results

The established one-step multiplex RT-qPCR method was applied to test 588 clinical samples. Among these samples, 91 were found to be positive for PRRSV, with a detection rate of 15.5% (91/588). Among them, 14 samples were positive for HP-PRRSV, with a detection rate of 2.4% (14/588); 3 samples were positive for C-PRRSV, with a detection rate of 0.5% (3/588); and 74 samples were positive for PRRSV-NADC30-like, with a detection rate of 12.59% (74/588). Parallel detection using the already reported RT-qPCR method showed that 76 samples were found to be positive for PRRSV, with a detection rate of 12.93% (76/588). Among them, 9 samples were positive for HP-PRRSV, with a detection rate of 1.53% (9/588); 2 samples were positive for C-PRRSV, with a detection rate of 0.34% (2/588); and 65 samples were positive for PRRSV-NADC30-like, with a detection rate of 11.05% (65/588). The Kappa value was 0.87, indicating almost perfect agreement between the two methods (Table 5). This supports the use of our developed method as an alternative or complementary approach for differential diagnosis of the three PRRSV subtypes in clinical samples. Sequencing of the amplified products with differences in the detection results between the two methods revealed that the reported detection method detected positive results because some base sequences had mutated. This indicates that updating the primer and probe sequences for RT-qPCR methods targeting different PRRSV strains is of great significance.

**TABLE 5:** Clinical sample testing and comparison with reported methods*.

This study established a method	The RT-qPCR method has been reported			
	Positive	Negative	In total	Accuracy rate (%)
Positive	74	17	91	81.31
Negative	2	495	497	99.60
In total	76	512	588	96.77

*Kappa = 0.87.

## 4 Discussion

PRRS, as one of the most economically destructive viral diseases in the global pig farming industry, has long severely restricted the healthy development of the industry. In China, this disease is mainly caused by PRRSV-2, and its clinical manifestations are complex and diverse, making prevention and control extremely difficult. Due to the inherent high variability of RNA viruses, various genotypes such as the classic strain, highly pathogenic strain, and NADC30-like strain have coexisted and frequently recombined in pig populations in China (Zhou et al., 2024). Accurate pathogen typing diagnosis is the prerequisite for formulating scientific prevention and control strategies (Li C. et al., 2025; Zhang et al., 2025). Therefore, establishing rapid, sensitive, and specific detection methods that can simultaneously identify and detect these three main epidemic strains has significant clinical significance.

The genetic variations of PRRSV, especially the highly variable regions of the *nsp2* gene, provide a molecular basis for the classification and identification of different strains (Zhao et al., 2022). Compared with the *nsp2* of the classical strain, highly pathogenic PRRSV strains have discontinuous 30aa (481aa, 534–562aa) deletions, while NADC30-like strains have discontinuous 131aa (322–432aa, 483aa, 504–522aa) deletions (Li H. et al., 2025; Wang C. et al., 2025). This makes the *nsp2* gene an ideal target for typing detection. However, due to the high variability of this region and the fact that NADC30-like strains are prone to mutations or recombination with other lineage strains, the sequence of the *nsp2* region shows high diversity (Li H. et al., 2025; Zhang et al., 2025). In this study, when designing primers and probes, we intentionally avoided the known recombination hotspots of the nsp2 gene (e.g., the deletion regions 322–432aa and 504–522aa, which are common recombination sites for NADC30-like strains). Instead, we selected relatively conserved regions flanking these hotspots for primer and probe binding, ensuring detection stability across different recombinant strains. If the design of primers and probes is based on an earlier reference sequence, as the prevalent strains continue to mutate, the matching degree between the detection target and the field-released strains will gradually decrease, resulting in false negative results. Therefore, continuous monitoring of the genetic sequence changes of prevalent strains, and regular updates of primers and probes for the fluorescence quantitative PCR method are crucial for the accuracy of clinical diagnosis.

This study downloaded a large number of PRRSV epidemic strain sequences from GenBank and combined with the genomic data of the latest epidemic strains isolated from the field by our laboratory. Through systematic comparison and analysis, three target regions that were conserved and type-specific for each strain were screened in the *nsp2* gene region. The primers and TaqMan MGB probes were updated, and a one-step multiplex RT-qPCR method for simultaneous detection of NADC30-like strains, highly pathogenic strains, and classic strains was successfully established. This method can differentiate and identify the three strains in a single reaction tube, featuring simple operation, high detection throughput, and low contamination risk. The method demonstrated excellent detection performance. The standard curve established for this method was within the range of 1 × 10^8^ to 1 × 10^3^ copies/μL. The correlation coefficients R^2^ for the detection of the three strains were all 0.998, and the amplification efficiencies were 98.38, 103.53, and 97.58%. This indicates that the method has excellent linear relationship and amplification efficiency. The minimum detection limits for C-PRRSV, PRRSV-NADC30-like, and HP-PRRSV were 10.128, 8.458, and 8.998 copies, respectively, with a sensitivity superior to or close to that of previously reported similar detection methods. For example, Ruan et al. established a four-step RT-qPCR detection method for simultaneously detecting PRRSV-1, classic strains of PRRSV-2, highly pathogenic strains, and NADC30-like strains, with a specificity of 97.33% and a sensitivity of 96.00% (Ruan et al., 2023). Tao et al. first designed primers and probes based on the M gene of PRRSV for the identification of PRRSV-1 and PRRSV-2, and further distinguished the 5 sublineages of PRRSV-2 based on the nsp2 gene: NADC30-like, NADC34-like, HP-PRRSV-like, VR-2332-like, and QYYZ-like. Finally, 7 sets of primers and probes were integrated into two sets of multiplex RT-qPCR methods. Comprehensive evaluation showed that the method had good repeatability (coefficient of variation CV < 2.12%), strong specificity, and the detection limits for the 7 detection targets were all lower than 5 copies/μL (Tao et al., 2024). It should be noted that the comparability of detection limits between our method and that of Tao et al. is influenced by differences in sample types, standard plasmid preparation, and reaction system conditions (e.g., reaction volume, cycling parameters); therefore, a direct superiority/inferiority conclusion based solely on the limit of detection values should be interpreted with caution. In addition, this method showed no cross-reaction with common swine pathogens such as PRRSV-1, PCV2, and CSFV, and had good repeatability and stability (CV < 2%).

Clinical sample testing revealed that the one-step triple RT-qPCR method developed in this study had a positive detection rate of 15.5% (91/588) for 588 clinical samples, while the parallel detection using the reported RT-qPCR method had a positive detection rate of 13.94% (82/288). The detection rate of this method was slightly higher than that of the reported method (Qiu et al., 2020). It is speculated that the samples not detected by the reported method were due to base mutations in the primer or probe binding regions, preventing the primers or probes from binding to the target sequence. This indicates that it is extremely important to update the primers and probes for the current prevalent strain sequence of PRRSV, and also reflects the severe challenge posed by PRRSV variation to the timeliness of diagnostic methods. Studies have shown that PRRSV-2 continuously undergoes mutations and recombination during its prevalence, especially the NADC30-like strain has become the dominant prevalent strain in China, and the sequence diversity of its Nsp2 and other target regions has been increasing (Zhang et al., 2025; Zhu et al., 2025). Zhang et al. analyzed the PRRSV prevalence in 27 provinces of China from 2024 to 2025 and found that non-recombinant NADC30-like strains have almost disappeared, and the vast majority of the prevalent strains have undergone recombination events (Zhang et al., 2025). The early established method was based on older reference sequences, and as the virus continues to evolve, it inevitably faces the risk of missed detection. Therefore, establishing a dynamic updated monitoring mechanism, regularly comparing the sequences of the detection targets and optimizing the primers and probes is a key measure to ensure the accuracy of PRRSV diagnosis.

Based on the typing results of clinical samples, among the 91 positive samples, the NADC30-like strains accounted for 81.31% of the positive samples, the HP-PRRSV accounted for 15.38%, and the C-PRRSV accounted for 3.3%. This detection result is consistent with the overall trend of the PRRSV epidemiological survey in China in recent years. Zhu et al. conducted a study on the prevalence of PRRSV in 27 provinces of China from 2024 to 2025, showing that currently, PRRSV-2 exists in 5 main lineages in China. Among them, lineage 1.8 (NADC30-like) accounted for 48.5% and was the dominant prevalent strain (Zhu et al., 2025). Li et al. conducted an epidemiological investigation of PRRSV in Sichuan Province from 2023 to 2024, indicating that lineage 1.8 (NADC30-like) was the main prevalent lineage in this region, and there was a complex situation of coexistence of multiple lineages such as lineage 8 (HP-PRRSV) and lineage 5 (classic strain) (Li M. et al., 2025). You et al. analyzed the recombinant strains of PRRSV in China from 2019 to 2023, also confirming that the NADC30-like strain has replaced the highly pathogenic strain as the dominant prevalent strain, and the recombination patterns mainly fall into two categories: those with NADC30 as the parent strain and those with HP-PRRSV as the parent strain (You et al., 2025). Additionally, Wang et al. conducted a molecular epidemiological survey of PRRSV in Henan and Shanxi provinces from 2023 to 2024, showing that the NADC30-like strain accounted for up to 77% and detected a recombination event between NADC30-like and VR2332-like (Wang T. et al., 2025). These research results are relatively consistent with the typing data of this study, all indicating that the NADC30-like strain remains the dominant prevalent strain in current pig herds in China.

Since the first report of the NADC30-like strain in China in 2012, with its strong recombination ability and adaptive advantages, it has gradually replaced the highly pathogenic strains and become the dominant prevalent strain. Studies have shown that the recombination events between NADC30-like strains and HP-PRRSV and other lineage strains have been increasing year by year, and the recombination hotspots are mainly distributed in the *nsp1, nsp4-nsp9* and ORF2-ORF6 regions. In this study, the highly pathogenic strain still accounted for 29.82% of the total, suggesting that HP-PRRSV still exists in a certain proportion of pig farms, especially in those without vaccine immunization or with unreasonable immunization programs. The highly pathogenic strain can still cause explosive epidemics (You et al., 2025). Although the detection rate of the classic strain is relatively low, as the “elder” strain of PRRSV, its genomic fragments may still exist in various recombination strains, and continuous monitoring of the prevalence of the classic strain is also of great significance for understanding the evolution pattern of the virus. In addition, no dual-positive signals were observed in the same reaction in this study, but Li et al.’s research in Sichuan region detected mixed infection of lineage 8 and PRRSV-1 in the same sample, suggesting that mixed infection does exist in some areas of China (Li M. et al., 2025). Considering the high-frequency recombination characteristics of the NADC30-like strain, the possibility of the presence of recombination strains in this study cannot be ruled out. Since the method established in this study is designed for the three prototype strains, for recombination strains, they may show double positivity or abnormal signal intensity, which provides important clues for the screening and identification of recombination strains in the future. However, the ability of this method to detect atypical recombinant strains or novel variants still requires further validation.

## 5 Conclusion

This study has updated and established a one-step multiplex RT-qPCR method capable of simultaneously detecting the classical strain, highly pathogenic strain, and NADC30-like strain of PRRSV. This method is highly sensitive, highly specific, has good repeatability, and has a good clinical application effect, providing a reliable technical tool for precise diagnosis and epidemiological monitoring of PRRSV.

## Data Availability

The original contributions presented in the study are included in the article/Supplementary material, further inquiries can be directed to the corresponding authors.
