# Supplementary material for: The updated one-step multiplex RT-qPCR method for PRRSV classical strains, highly pathogenic strains and NADC30-like strains
Source: Front Microbiol. 2026 Jun 11;17:1850527. doi: 10.3389/fmicb.2026.1850527 (PMC13294042; doi:10.3389/fmicb.2026.1850527)
Supplement: Supplementary file 1 [file Supplementary_file_1.pdf]

Table S1 The detection rates and average Ct values of different gradient positive nucleic acid particles

Recombinant plasmids	Concentrations (copies/μL)	samples	the one-step multiplex RT-qPCR method	
			Ct (average)	Detection rate (%)
C-PRRSV	20	25	38.11	100
	10	25	38.87	92
	5	25	Undetermined	0
	1.25	25	Undetermined	0
PRRSV-NADC30-like	20	25	35.66	100
	10	25	36.82	100
	5	25	37.55	8
	1.25	25	Undetermined	0
HP-PRRSV	20	25	37.13	100
	10	25	38.49	100
	5	25	Undetermined	0
	1.25	25	Undetermined	0

Table S2 Regression analysis of the minimum detectable value of the classical PRRSV strain

Probit	The 95% confidence limit of the minimum detectable value		
	Estimate	Lower limit	Upper limit
0.01	5.527	-1.251	7.223
0.02	5.843	-0.431	7.429
0.03	6.043	0.088	7.561
0.04	6.194	0.477	7.66
0.05	6.316	0.794	7.742
0.06	6.421	1.063	7.812
0.07	6.512	1.299	7.873
0.08	6.594	1.51	7.928
0.09	6.669	1.701	7.979
0.1	6.737	1.878	8.025
0.15	7.021	2.605	8.22
0.2	7.247	3.18	8.378
0.25	7.441	3.67	8.516
0.3	7.614	4.108	8.643
0.35	7.776	4.512	8.763
0.4	7.928	4.891	8.88
0.45	8.076	5.255	8.996
0.5	8.222	5.61	9.114
0.55	8.368	5.96	9.237
0.6	8.515	6.31	9.368
0.65	8.668	6.664	9.51
0.7	8.829	7.026	9.67

0.75	9.003	7.403	9.858
0.8	9.197	7.8	10.09
0.85	9.423	8.227	10.397
0.9	9.707	8.697	10.848
0.91	9.775	8.8	10.968
0.92	9.85	8.906	11.104
0.93	9.932	9.017	11.259
0.94	10.023	9.135	11.438
**0.95**	**10.128**	**9.262**	**11.65**
0.96	10.25	9.401	11.909
0.97	10.401	9.561	12.239
0.98	10.601	9.756	12.694
0.99	10.917	10.035	13.441

Table S3 Regression analysis of the minimum detectable value of PRRSV NADC30-like strains

	The 95% confidence limit of the minimum detectable value		
**Probit**	**Estimate**	**Lower limit**	**Upper limit**
0.01	4.075	-1.044	4.91
0.02	4.376	0.433	5.177
0.03	4.567	1.336	5.38
0.04	4.71	1.986	5.562
0.05	4.827	2.488	5.736
0.06	4.926	2.89	5.91
0.07	5.014	3.219	6.087
0.08	5.092	3.491	6.267
0.09	5.163	3.718	6.451
0.1	5.228	3.91	6.638
0.15	5.499	4.53	7.586
0.2	5.714	4.868	8.493
0.25	5.898	5.092	9.338
0.3	6.064	5.262	10.128
0.35	6.217	5.401	10.879
0.4	6.363	5.522	11.602
0.45	6.504	5.632	12.308
0.5	6.643	5.734	13.01
0.55	6.781	5.833	13.715
0.6	6.922	5.929	14.435
0.65	7.068	6.027	15.181
0.7	7.221	6.127	15.971
0.75	7.387	6.232	16.825
0.8	7.572	6.348	17.778
0.85	7.787	6.48	18.892
0.9	8.057	6.644	20.296

0.91	8.123	6.683	20.636
0.92	8.194	6.725	21.005
0.93	8.272	6.771	21.41
0.94	8.359	6.823	21.864
**0.95**	**8.458**	**6.882**	**22.381**
0.96	8.575	6.951	22.989
0.97	8.719	7.035	23.737
0.98	8.91	7.146	24.732
0.99	9.21	7.32	26.301

Table S4 Regression analysis of the minimum detectable value of the HP-PRRSV strain

	The 95% confidence limit of the minimum detectable value		
**Probit**	**Estimate**	**Probit**	**Estimate**
0.01	5.378	0.969	6.987
0.02	5.626	1.569	7.214
0.03	5.784	1.943	7.364
0.04	5.902	2.221	7.48
0.05	5.999	2.444	7.578
0.06	6.081	2.632	7.663
0.07	6.153	2.796	7.739
0.08	6.218	2.941	7.808
0.09	6.276	3.071	7.873
0.1	6.33	3.191	7.933
0.15	6.554	3.674	8.193
0.2	6.731	4.044	8.413
0.25	6.884	4.35	8.614
0.3	7.02	4.616	8.803
0.35	7.147	4.854	8.986
0.4	7.268	5.072	9.168
0.45	7.384	5.276	9.351
0.5	7.499	5.469	9.539
0.55	7.613	5.656	9.733
0.6	7.729	5.838	9.938
0.65	7.85	6.019	10.157
0.7	7.977	6.201	10.396
0.75	8.113	6.389	10.663
0.8	8.266	6.588	10.971
0.85	8.443	6.808	11.342
0.9	8.667	7.066	11.826
0.91	8.721	7.126	11.946
0.92	8.78	7.19	12.077
0.93	8.844	7.259	12.222
0.94	8.916	7.335	12.386
**0.95**	**8.998**	**7.419**	**12.574**

0.96	9.095	7.516	12.798
0.97	9.213	7.633	13.076
0.98	9.371	7.782	13.451
0.99	9.619	8.008	14.052
